# The first mitochondrial genome for the subfamily Asopinae (Heteroptera: Pentatomidae) and its phylogenetic implications

**DOI:** 10.1080/23802359.2017.1398599

**Published:** 2017-11-09

**Authors:** Qing Zhao, Jiufeng Wei, Wanqing Zhao, Bo Cai, Xin Du, Hufang Zhang

**Affiliations:** a Department of Entomology, Shanxi Agricultural University, Taigu, China;; b Hainan Entry-Exit Inspection and Quarantine Bureau, Haikou, Hainan, China;; c Institute of Biomedical Sciences, College of Life Sciences, Shandong Normal University, Jinan, China

**Keywords:** Heteroptera, Pentatomidae, Asopinae, *Picromerus griseus*, mitogenome

## Abstract

Here, we determined the complete mitogenome of *Picromerus griseus*, the first for the subfamily Asopinae. This mitochondrial genome contains 16,338 bp, with an A + T content of 71.69%, and contains 37 typical mitochondrial genes (13 protein-coding genes, 22 transfer RNA genes, two ribosomal RNA genes and a control region). The genome size, gene arrangement, A + T content, codon usage and secondary structures of 22 transfer RNA genes of the *P. griseus* mitogenome were similar to those of other sequenced pentatomoids. Bayesian analyses performed using the mitogenomes of *P. griseus* and its relatives, including 14 taxa, confirmed the reasonable placement of *P. griseus*.

Asopinae (Insecta: Heteroptera: Pentatomomorpha: Pentatomidae) is a subfamily of Pentatomidae, which is a large predatory stink bug that usually feeds on the larvae of various Coleoptera, Lepidoptera and Hemiptera. To date, all the pentatomid mitogenomes (mt-genomes) are from Pentatominae and Podopinae, which limits our understanding of the diversity and phylogeny of Pentatomidae (Wang et al. 2017). Herein, we report and analyze the complete mt-genome of *Picromerus griseus*, which is commonly used in biological control strategies. Adults specimens of *P. griseus* were collected from Baoshan City (25.3 N, 98.8 E), Yunnan Province, China, on 15 August 2015. Both the voucher specimens and remaining genomic DNA were deposited in the Institute of Entomology, Shanxi Agricultural University.

The complete mt-genome of *P. griseus* is a double-stranded circular molecule that is 16,338 bp long (GenBank accession number: MF805778) and contains 37 typical mitochondrial genes: 13 protein-coding genes (PCGs), 22 transfer RNA genes (tRNAs), the large and small ribosomal RNA unit genes (rrnL and rrnS) and a control region. The gene order and orientation of the mitochondrial genes are identical to those of most other true bugs (Hua et al. 2009), which was considered to be the ancestral arrangement.

The nucleotide composition of the *P. griseus* mt-genome is A 39.26%, G 11.79%, C 16.53% and T 32.42%, showing a significant bias towards A and T of 71.68%.

Most PCGs share the start codons of ATN (four with ATA, four with ATG and four with ATT), except that COI starts with TTG. This unconventional start codon has also been reported for some other heteropterans (Hua et al. 2008; Zhao et al. 2017). Ten PCGs share the same termination codon of TAA, two PCGs (ND5 and ND6) end with TAG and COII is terminated with a single T.

The 22 tRNAs range from 61 to 76 bp, and all have a typical cloverleaf secondary structure except trnS-*Ser* (GCT) and trnV-*Val* (TAC), which lack a dihydrouridine arm. The rrnL has a length of 1302 bp with an A + T content of 75.73%, while rrnS has a length of 807 bp with an A + T content of 75.22%, and they are separated from each other by trnV-*Val*. The D loop region is located between the 12S rRNA and trnI-*Ile* with a total length of 1521 bp.

Phylogenetic analyses of 14 superfamily Pentatomoidea species and two superfamily Coreoidea species were conducted using Bayesian inference on 14 nucleotide sequences of 13 mitochondrial PCGs, resulting in a tree topology (Figure 1). Bayesian analyses were conducted using GPU MrBayes (Zhou et al. 2011) under the GTR + I + G model estimated by PartitionFinder v1.1.0 (Lanfear et al. 2012). Each family formed a monophyletic cluster with a high degree of bootstrap support. In Pentatomidae, the species *G. rubrolineata*, which belongs to the subfamily Podopinae, was separated from two other subfamilies. Although *P. griseus* was from the subfamily Asopinae, this species mixed with species from the subfamily Pentatominae. This suggested that Pentatominae might not be monophyletic or that Asopinae was more closely related with Pentatominae. Because mt-genome sequences are limited, the sequencing of more mt-genomes is needed to adequately resolve the subfamily relationships within Pentatomidae, and more mt-genome sequences are required to resolve the family relationships within Pentatomoidea. Therefore, further studies are needed to sequence more species from Asopinae and other subfamilies, which will enhance our understanding of the molecular phylogeny in Pentatomidae. This is the first sequenced complete mt-genome from the subfamily Asopinae, and the mitogenomic data of *P. griseus* will help to better understand the population genetics and evolution of Pentatomoidea.

**Figure 1.**
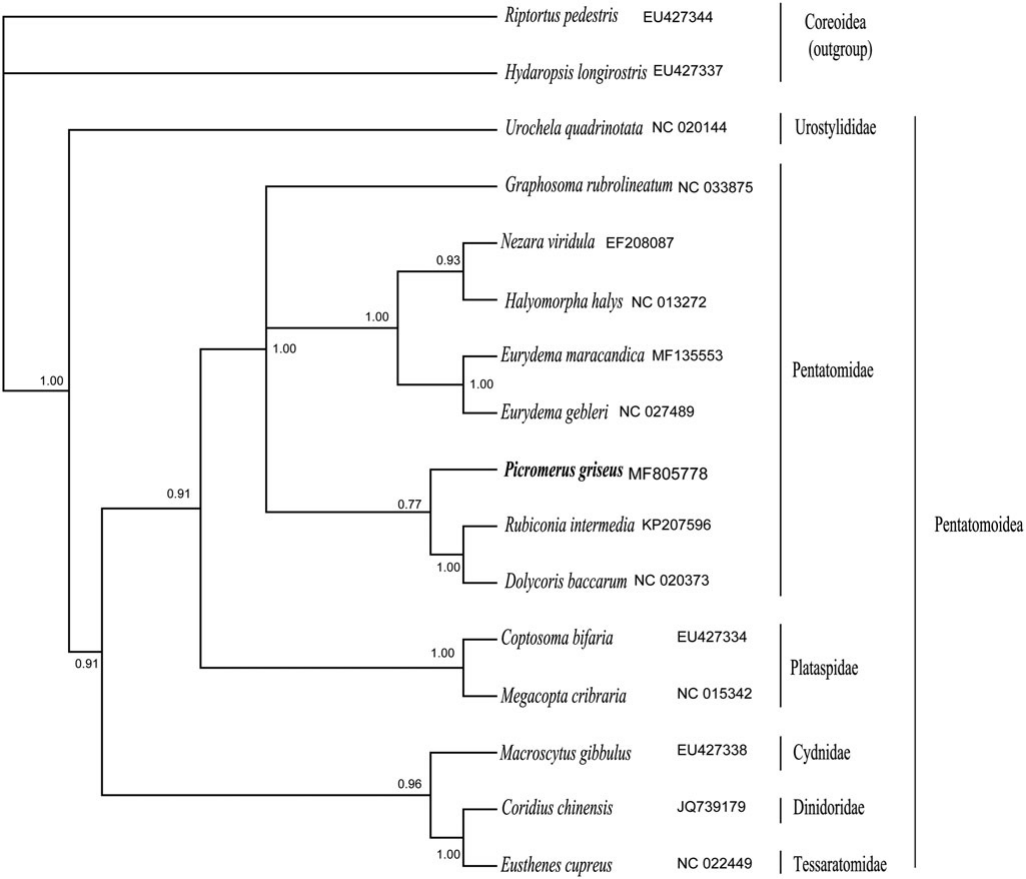
Phylogenetic relationship of *P. gresius* within Pentatomoidea inferred from 13 PCGs. Numbers on branches are Bayesian posterior probabilities.

## References

[CIT0001] Hua JM, Li M, Dong PZ, Cui Y, Xie Q, Bu WJ. 2008 Comparative and phylogenomic studies on the mitochondrial genomes of Pentatomomorpha (Insecta: Hemiptera: Heteroptera). BMC Genomics. 9:610. 19091056 10.1186/1471-2164-9-610 PMC2651891

[CIT0002] Hua JM, Li M, Dong PZ, Cui Y, Xie Q, Bu WJ. 2009 Phylogenetic analysis of the true water bugs (Insecta: Hemiptera: Heteroptera: Nepomorpha): evidence from mitochondrial genomes. BMC Evol Biol. 9:134. 19523246 10.1186/1471-2148-9-134 PMC2711072

[CIT0003] Lanfear R, Calcott B, Ho SY, Guindon S. 2012 Partitionfinder: combined selection of partitioning schemes and substitution models for phylogenetic analyses. Mol Biol Evol. 29:1695–1701. 22319168 10.1093/molbev/mss020

[CIT0004] Wang J, Zhang L, Yang XZ, Zhou MQ, Yuan ML. 2017 The first mitochondrial genome for the subfamily Podopinae (Hemiptera: Pentatomidae) and its phylogenetic implications. Mitochondrial DNA B Resour. 24:219–220. 10.1080/23802359.2017.1310605 PMC7800415 33473775

[CIT0005] Zhao WQ, Zhao Q, Li M, Wei JF, Zhang XH, Zhang HF. 2017 Characterization of the complete mitochondrial genome and phylogenetic implications for *Eurydema maracandica* (Hemiptera: Pentatomidae). Mitochondrial DNA B Resour. 2:219–220. 10.1080/23802359.2017.1365649 PMC7800374 33490465

[CIT0006] Zhou JF, Liu XG, Stones DS, Xie Q, Wang G. 2011 MrBayes on a graphics processing unit. Bioinformatics. 27:1255–1261. 21414986 10.1093/bioinformatics/btr140

